# Automation of generative adversarial network-based synthetic data-augmentation for maximizing the diagnostic performance with paranasal imaging

**DOI:** 10.1038/s41598-022-22222-z

**Published:** 2022-10-27

**Authors:** Hyoun-Joong Kong, Jin Youp Kim, Hye-Min Moon, Hae Chan Park, Jeong-Whun Kim, Ruth Lim, Jonghye Woo, Georges El Fakhri, Dae Woo Kim, Sungwan Kim

**Affiliations:** 1 Transdisciplinary Department of Medicine and Advanced Technology, Seoul National University Hospital, Jongno-Gu, Seoul, 03080 Republic of Korea; 2 Medical Big Data Research Center, Seoul National University College of Medicine, Jongno-Gu, Seoul, 03080 Republic of Korea; 3 Department of Biomedical Engineering, Seoul National University College of Medicine, 101 Daehak-Ro, Jongno-Gu, Seoul, 03080 Republic of Korea; 4 Department of Otorhinolaryngology-Head and Neck Surgery, Ilsan Hospital, Dongguk University, Gyeonggi, 10326 Republic of Korea; 5 Interdisciplinary Program of Medical Informatics, Seoul National University College of Medicine, Seoul, 03080 Republic of Korea; 6 Interdisciplinary for Bioengineering, Seoul National University, Jongno-Gu, Seoul, 03080 Republic of Korea; 7 Department of Otorhinolaryngology-Head and Neck Surgery, Seoul National University Bundang Hospital, Gyeonggi, 13620 Republic of Korea; 8 Department of Radiology, Massachusetts General Hospital and Harvard Medical School, Boston, MA 02114 USA; 9 Department of Otorhinolaryngology-Head and Neck Surgery, Boramae Medical Center, Seoul Metropolitan Government-Seoul National University 20, Boramae-Ro 5-Gil, Dongjak-Gu, Seoul, 07061 Republic of Korea; 10 Department of Biomedical Engineering, Seoul National University Hospital, Jongno-Gu, Seoul, 03080 Republic of Korea

**Keywords:** Biomedical engineering, Medical imaging

## Abstract

Thus far, there have been no reported specific rules for systematically determining the appropriate augmented sample size to optimize model performance when conducting data augmentation. In this paper, we report on the feasibility of synthetic data augmentation using generative adversarial networks (GAN) by proposing an automation pipeline to find the optimal multiple of data augmentation to achieve the best deep learning-based diagnostic performance in a limited dataset. We used Waters’ view radiographs for patients diagnosed with chronic sinusitis to demonstrate the method developed herein. We demonstrate that our approach produces significantly better diagnostic performance parameters than models trained using conventional data augmentation. The deep learning method proposed in this study could be implemented to assist radiologists in improving their diagnosis. Researchers and industry workers could overcome the lack of training data by employing our proposed automation pipeline approach in GAN-based synthetic data augmentation. This is anticipated to provide new means to overcome the shortage of graphic data for algorithm training.

## Introduction

Recent developments in deep learning suggest the possibility of using medical imaging for artificial intelligence (AI)-assisted diagnosis. Compared with traditional machine learning, deep learning requires larger labeled datasets to achieve sufficient model performance^1–4^. Collecting numerous real healthcare datasets for effective deep learning performance is generally complex and time-consuming since the labeling process relies on the radiologists’ experience and is limited by patients’ privacy concerns^1,2,5^. Furthermore, model training is often difficult to perform in primary or secondary hospitals due to a lack of training datasets. Therefore, deep learning research and commercialization in health care institutions often face limiting obstacles. Data augmentation techniques have been widely used to overcome the limited data problem. Common data augmentation methods for medical image analysis include simple geometric and intensity transformation^6–9^. However, when using these conventional methods, little additional information is obtained through small modifications made to original images, which only partially improves model performance.

The generative adversarial netw**o**rks (GAN)^10^ technique is another alternative solution that has been successfully applied to deep learning in the case of a limited dataset. GAN employs neural networks to create generative models. It is commonly used for synthetic data augmentation as it can generate plausible new images from unlabeled original ones^11,12^. Several prior studies have reported the application of various GAN frameworks to overcome the challenges of insufficient training datasets and enhance the algorithm’s performance to efficiently assist radiologists in diagnosing different types of diseases through medical imaging data^13–18^.

When conducting data augmentation, the suitable augmented sample size for optimizing model performance is usually unknown. Reported works pertaining to the generation of medical data using GAN rarely provide specific explanations regarding the amount of data they decided to produce. The augmented sample sizes were increased randomly without specific rules, even though a previous study investigated the effect of sample size in data augmentation on the performance of the model and defined the optimal point of the synthetic data augmentation at the highest accuracy score^19^.

In this study, we propose an automation pipeline for GAN-based synthetic data augmentation by adopting the concept of sliding window to select the ideal number of samples needed for the best model that improves the diagnosis performance of the deep learning algorithm in the presence of a limited dataset. We used paranasal (PNS) radiographs for our experimental data, therefore, acquired PNS x-ray images from a secondary hospital to build our deep learning model. To the best of our knowledge, this study is, clinically, the first to report the feasibility of generating the synthetic data using GAN with paranasal radiography. We subsequently evaluated the model using an additional independent test set from a tertiary hospital to demonstrate that synthetic data augmentation using our approach automation pipeline allows effective algorithm training that can achieve more generalization in primary or secondary hospital with a relatively small number of datasets.

## Results

We analyzed the performance of the ChexNet model, which is a convolutional neural network trained using 112,120 Chest X-ray images^20^, through four different experiments: (1) CheXNet model trained only with original training data (OD); (2) CheXNet model trained with GAN-based class balancing method (GCB); (3) CheXNet model trained with GAN-based class balancing and conventional data augmentation method (GCB + CDA); 4) CheXNet model selected by the suggested automation pipeline, which is the trained model of K_optimal_ (GCB + OMGDA). All experiments were evaluated with the internal test set (sinusitis = 43, healthy sinus = 36).

### Finding an optimal multiple number of the synthetic data augmentation

**Figure 1:**
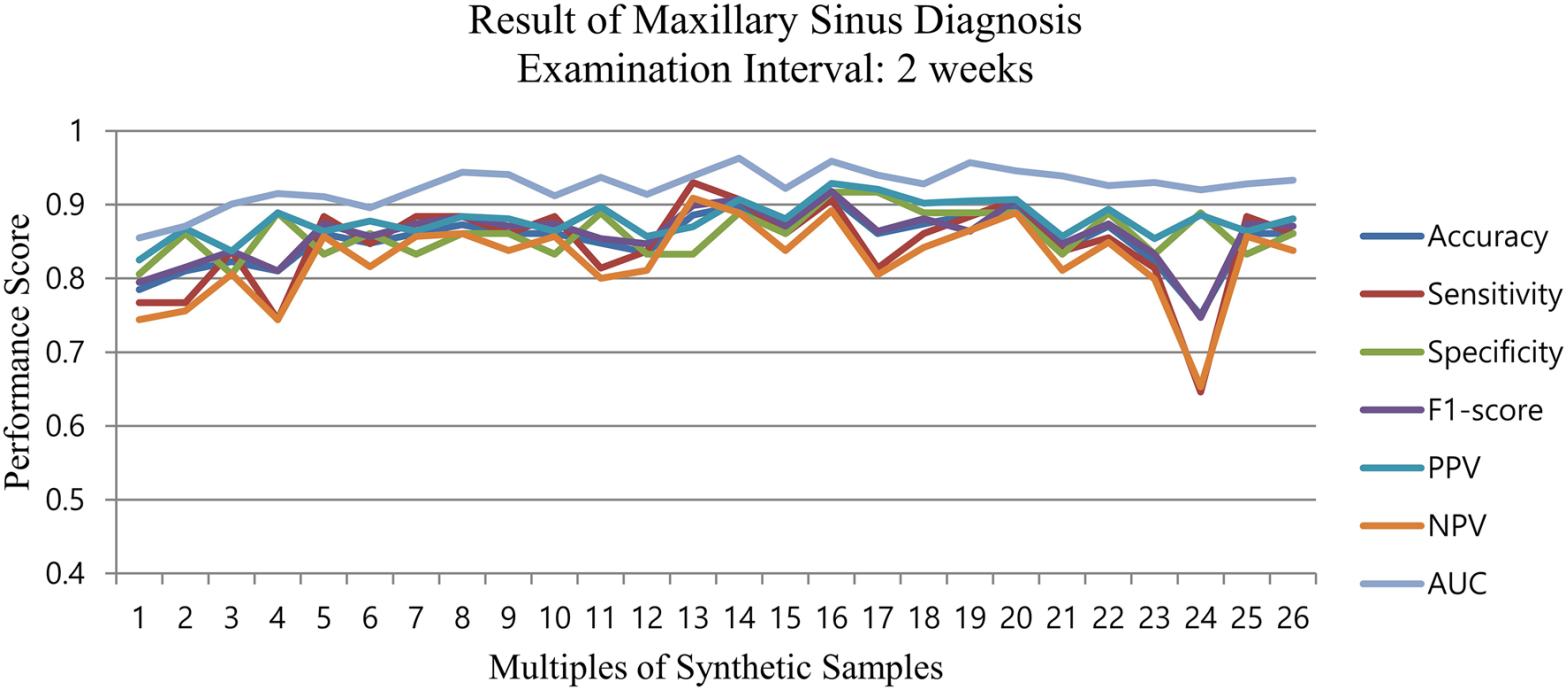
CheXNet performance with an increase in the synthetic training sample size.

The automation pipeline was proposed to find the optimal multiple number (K_optimal_) of the synthetic data augmentation using the sliding window method which is described in the METHOD. The highest diagnostic performance was found for K_optimal_ = 14 based on the area under the curve (AUC) score. Furthermore, the stopping point of the sliding window was achieved when the synthetic data augmentation was conducted up to a factor of 26 (n = 7904). Supplementary Table S1 represents the effect of increasing the number of synthetic training samples for synthetic data augmentation on the classification performance. It was observed that the AUC of classification gradually increased and achieved its top performance at 4256 training samples (K_optimal_ = 14) with an accuracy of 0.899, sensitivity of 0.907, specificity of 0.889, F1-score of 0.907, PPV of 0.907, NPV of 0.889, and AUC of 0.963. Subsequently, the AUC kept fluctuating around 0.93 and did not show any further improvement. Figure 1 shows the scatter plot of all performances based on the size of training samples.

### Performance evaluation for PNS X-ray classification

Table 1 exhibits the results of the performance evaluation of the four different models using the internal test set.

**Table 1:** Performance evaluation of internal test set for CheXNet model.

Performance metrics for model predictions	OD^a^	GCB^b^	GCB + CDA^c^	GCB + OMGDA^d^
Accuracy	0.785	0.798	0.81	0.899
Sensitivity	0.767	0.861	0.791	0.907
Specificity	0.806	0.722	0.833	0.889
F1-score	0.795	0.822	0.819	0.907
PPV	0.825	0.787	0.850	0.907
NPV	0.744	0.813	0.769	0.889
AUC	0.855	0.887	0.908	0.963

^a^CheXNet model trained using original data, only.

^b^CheXNet model trained with GAN-based class balancing.

^c^CheXNet model trained with GAN-based class balancing and conventional data augmentation.

^d^CheXNet model trained with the output of the suggested automation pipeline, which is the trained model of K_optimal_ (GCB + OMGDA).

**Figure 2:**
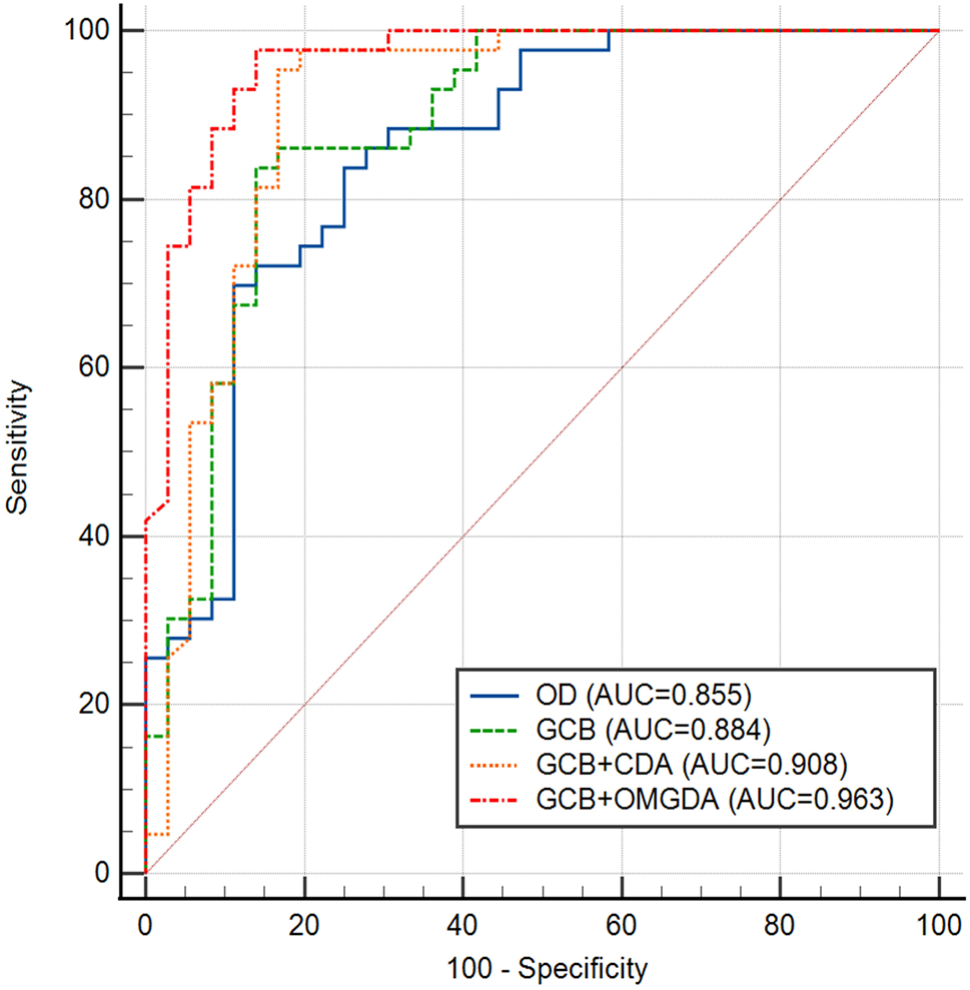
Receiver operating characteristic (ROC) curve analysis of four experimental models using the internal test set. (GCB: GAN-based class balancing; CDA: conventional data augmentation; OMGDA: optimal multiple of GAN-based data augmentation).

The screening performance of the CheXNet model with conventional data augmentation after class balancing (GCB + CDA) showed results that were better than those of the two remaining models (OD and GCB) but worse than those of our proposed model. The ROC curve analysis for each of the four experimental models using the internal test set is shown in Fig. 2.

Our proposed model showed a higher AUC than the OD and GCB models (*P* = 0.018 and *P* = 0.021) for model performance evaluation using the internal test set. It was observed that the CheXNet model with conventional data augmentation (GCB + CDA) did not show any statistical difference compared to the OD or GCB models. However, GAN-based data augmentation (GCB + OMGDA) showed statistical significance compared with the other models.

We further evaluated the GCB + OMGDA model which was selected by our proposed automation pipeline using the external test set (sinusitis = 66, healthy sinus = 66), as it had the highest AUC performance among the other models, OD, CBD, and CB + CDA. The performance evaluation of GCB + OMGDA using the external test set showed an accuracy of 0.833, sensitivity of 0.879, specificity of 0.788, F1-score of 0.841, PPV of 0.810, NPV of 0.867, and AUC of 0.924. This indicates that the ACGAN and its variants can be successfully used for data augmentation and for generating synthetic data that are as good as original images.

## Discussion

In the present study, we enrolled 195 subjects with a maximum interval of 14 days between the radiographic examination and CT for the internal dataset. In tertiary hospitals, PNS CT was often used as a diagnostic tool for these patients instead of PNS radiographs because most patients were already diagnosed with chronic rhinosinusitis (CRS) from primary clinics. Nonetheless, PNS radiography was performed in cases with uncertain diagnosis or symptomatic improvement. Thus, few patients underwent both examinations within an interval of 2 weeks, and 195 subjects did not represent a sufficient number for the application of deep learning.

The data augmentation technique has been used widely to enhance the model performance using a limited amount of training data. However, the optimal multiple of augmented data needed to train the deep learning model showing the best performance remains unknown. We propose the use of GAN-based synthetic data augmentation to improve the classification of PNS radiographs using the transfer learning method to overcome the aforementioned challenge. An automatic approach to investigate the optimal multiple numbers of synthetic data augmentation was proposed using the sliding window method.

Using 389 X-ray images to diagnose CRS, we found that the classification model shows the best diagnostic performance when trained with the original data, together with GAN-generated synthetic data amounting to 13-times the original data. The optimal multiple of synthetic data required to best train the model should depend on the type and size of the original data. However, our method was shown to help find the optimal amount of synthetic data to be used for deep learning model training under different circumstances.

According to the synthetic data augmentation results, it was observed that there was no remarkable improvement in the performances after increasing the synthetic data size further than a factor of 14 from the original training data. A possible explanation for this observation could be the mode collapse of the ACGAN model. Mode collapse is a common problem faced in GAN model training where the generator collapses, producing a limited variety of samples. We quantitatively measured image similarities using multi-scale structural similarity (MS-SSIM)^21,22^ to evaluate the potential mode collapse. We observed the changes in the MS-SSIM scores of synthetic images for factors beyond 14 (see Supplementary Fig. S2 for details) based on the synthetic image datasets. MS-SSIM attempts to discount aspects of the image that are not important for human perception. MS-SSIM outputs the value range between [0, 1]. Higher values correspond to perceptually more similar images, and lower values represent better image diversity. We randomly selected 100 pairs of images from the real training set and the ACGAN-based synthetic images of the clear sinus and sinusitis classes. For synthetic data, we observed the changes in the MS-SSIM scores of synthetic images for factors beyond 14. As a result, all the MS-SSIM scores of the synthetic images fluctuated at around 0.5, which is comparable to the MS-SSIM score of the original images. This indicates that model collapse did not appear within our ACGAN model.

In a previous related study, the augmentation sample sizes were increased with no specific rule. Furthermore, no clearly defined standards regarding when to stop the data augmentation were thus available^19^. Our proposed method automatically finds the optimal multiple numbers of synthetic data augmentation at the highest model performance by using the sliding window. Consequently, our proposed method is more quantitative and less labor-intensive. The proposed method was applied solely to synthetic data augmentation, which showed the effects of GAN with high diagnostic performance.

Nevertheless, the present work has several limitations. Ideally, only the subjects who underwent PNS conventional radiography and CT on the same day should have been enrolled. However, there were only 40 such subjects. Therefore, we extended the examination interval to 14 days, excluding patients with acute aggravation of the disease in this interval. Moreover, ACGAN was arbitrarily chosen and applied in this study to develop the automation pipeline since it is known and used widely for generating high-resolution images; however, higher performance could also be obtained using other GAN techniques. Furthermore, all conventional data augmentation methods were not used; only a slight modification (horizontal flip and rotation within − 10 to 10°) was applied in this study. The diagnostic performance using conventional data augmentation could be improved further by applying additional methods such as intensity transformation (e.g., brightness, contrast, and noise). Lastly, we conducted the external validation with datasets from another medical institution (tertiary hospital). The inherent difference in the quality of medical datasets owing to the characteristics of their source (e.g., physicians’ diagnostic skills and equipment type and quality) could have affected the results^23^. Thus, additional external validations using datasets acquired from various institutions are warranted to compensate for this potential bias and generalize our findings.

In conclusion, our proposed GAN-based synthetic data augmentation performed well for the diagnosis of maxillary sinusitis, despite a small sample size. The deep learning approach proposed in this study could assist radiologists in improving diagnosis. Moreover, researchers and industry workers could overcome the lack of training data by using our approach for GAN-based synthetic data augmentation. Thus, even personnel from primary clinics and secondary hospitals could effectively train deep learning models with adequate performance using their own datasets.

## Methods

This retrospective study was approved by the institutional review board of the Seoul National University Boramae Medical Center (IRB No. 10-2020-173) and Seoul National University Bundang Hospital [IRB No. B-2010-645-401] and was performed in accordance with the Declaration of Helsinki.

### Datasets

**Figure 3:**
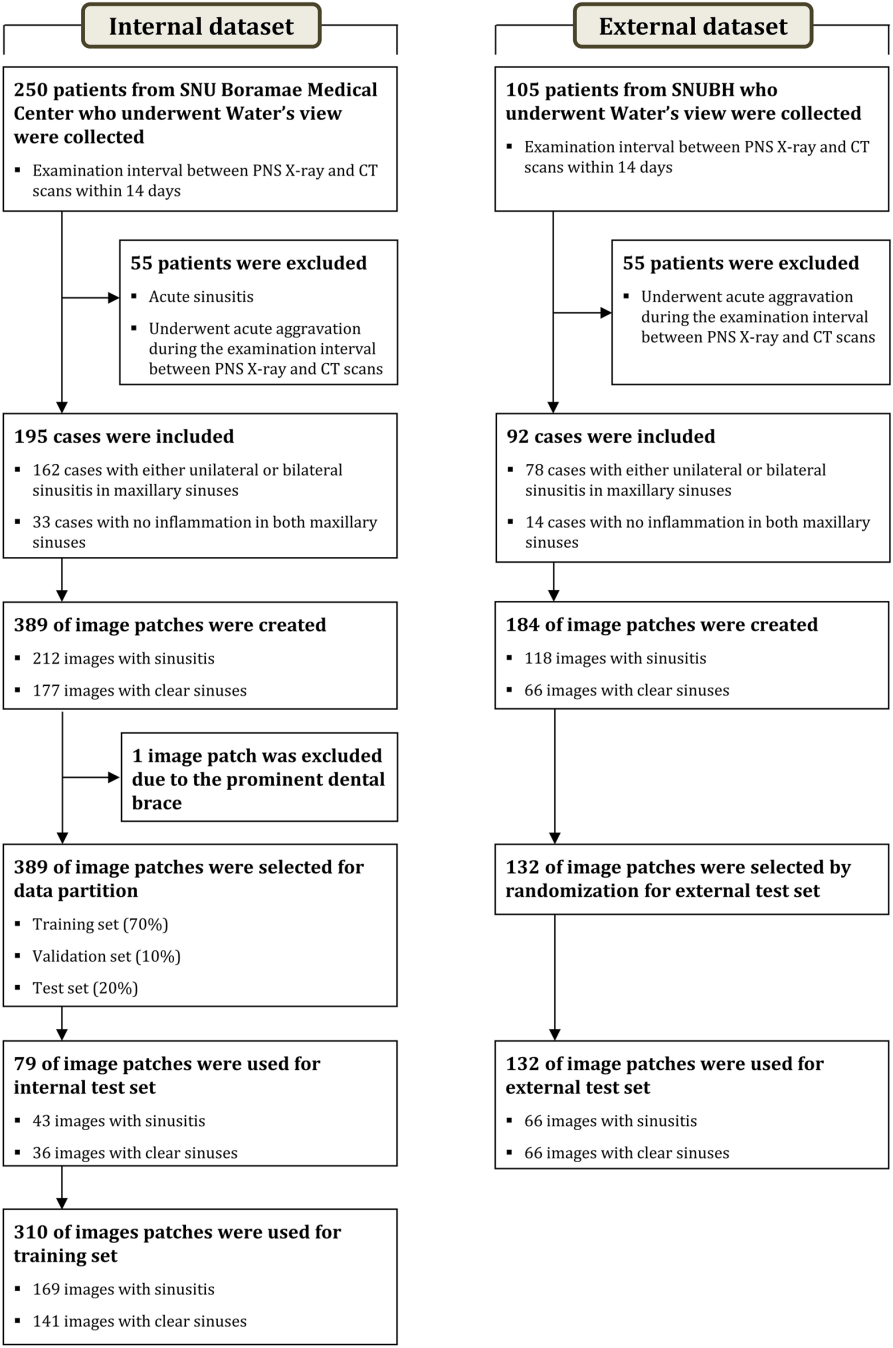
Flowchart of the inclusion and exclusion criteria of this study.

PNS radiographs, obtained using the Waters’ view, were acquired for 355 patients divided into two sets (250 patients for the internal dataset and 105 for the external dataset). Patients were diagnosed with chronic sinusitis (CRS) at the Seoul National University Boramae Medical Center (for the internal dataset) and Seoul National University Bundang Hospital (for the external dataset). They all underwent radiographic examination between January 2010 and January 2020 (internal dataset) and between August 2003 and April 2019 (external dataset). We included only those patients who underwent conventional radiographic examination and paranasal CT scans within 14 days (mean interval: 6 days) of each other. Patients with acute sinusitis and those who underwent acute aggravation during the examination interval between conventional radiography and CT scans were excluded. The design for the present study is shown in Fig. 3. All findings from conventional radiographs were independently labeled as “normal” or “sinusitis” by an expert rhinologist. These findings were verified using the PNS CT scans (clinical explanation on datasets is included in the Supplementary Material).

### Data preparation

**Figure 4:**
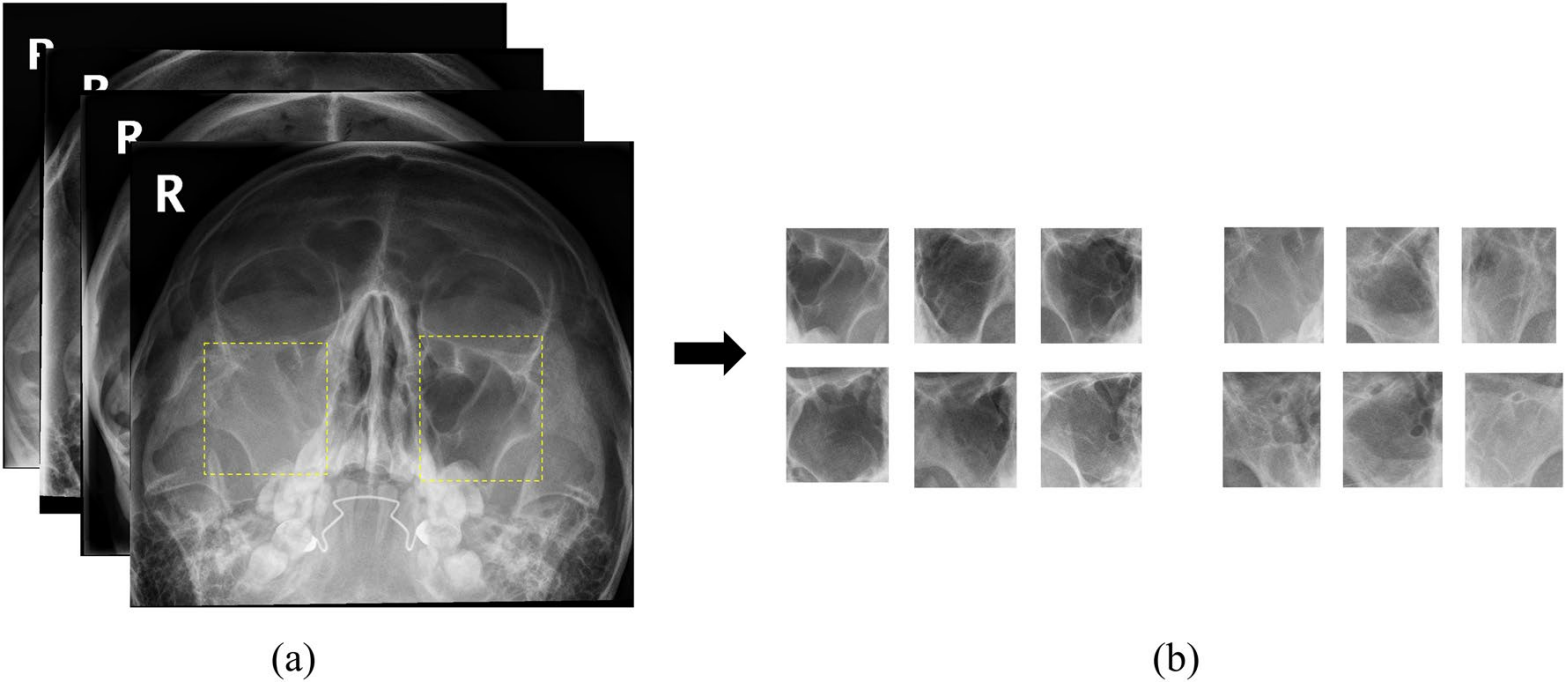
Regions of interest (ROI) on Water's view of paranasal radiographs. (**a**) Original image with ROI annotation. (**b**) Cropped images of clear sinuses (left) and inflamed sinuses (right).

During pre-processing, the region of interest (ROI) was annotated based on the anatomical structure of the maxillary sinus reviewed by a rhinologist (Fig. 4). The respective ROIs of the bilateral maxillary sinuses were cropped as image patches with MATLAB (2019a) and used for model training to enhance the training performance. The size of the image patches varied based on the size of the maxillary sinus. Image patches of 212 inflammatory and 177 healthy sinuses were created for the internal dataset and of 118 inflammatory and 66 healthy sinuses for the external test set in a similar manner. The class distributions of the internal dataset and external test set were 1:1.2 and 1:1.8, respectively.

The internal dataset was divided into a training set (70%), a tuning set set (10%), and a test set (20%) using stratified random sampling to maintain the class ratio between the training and test sets (Table 2).

**Table 2:** The composition of internal paranasal X-ray dataset.

Data class	Number of ROI patches		
	Healthy sinus	Sinusitis	Total
Training set (70%)	127	152	279
Tuning set (10%)	14	17	31
Test set (20%)	36	43	79
Total	177	212	389

### Image generation using auxiliary classifier GAN

GAN has been widely used for data augmentation due to its hybrid model technique invented by Goodfellow in 2014^10^. It has the advantage of providing a way to learn deep learning representation with less annotated training samples. GAN is an algorithmic architecture consisting of two neural networks: the first is a generator focusing on image generation, while the second focuses on discrimination. By alternating these two networks, the generator learns to construct samples similar to real images and the discriminator gets to differentiate between true and false data. A random vector noise $z$ is used as an input to the generator ($G$), which produces samples $G(z)$ that have a similar distribution to that of the training set (original images). The discriminator $D$ attempts to learn to distinguish generated samples from real ones. The adversarial competition between the generator and the discriminator is followed by the concept of a two-play minimax game described by

1 $$\mathop {\min }\limits_{G} \mathop {\max }\limits_{D} V(D,G) = E_{{x\sim P_{data} (x)}} \left[ {\log D(x)} \right] + E_{z\sim Pz(z)} [\log (1 - D(G(z)))]$$

where $P_{data}$ and $P_{z} (z)$ are the probability distributions for the original samples and generated samples, respectively. In the objective function, the discriminator is trained to maximize $D(x)$ for images with $x$ ~ $P_{data}$, and the generator produces images $G(z)$ to fool $D$ during training (i.e.,$D(G(z))$ ~ $P_{data}$). Intuitively, the generator tries to fool the discriminator to the best of its ability by generating samples that look indistinguishable from $P_{data}$, while the discriminator improves its ability to distinguish real images from synthesized ones.

In this work, we used an advanced version of GAN called Auxiliary Classifier GAN (ACGAN)^21^ to perform synthetic data augmentation. Conditional GAN (CCAN)^24^ was also introduced to improve the sample quality (e.g., resolution) of the generative model by allowing the model to be conditioned on external information. ACGAN is a type of CGAN that allows the discriminator to be tasked with reconstructing side information, such as predicting an image’s class label instead of receiving it as an input. It applies the associated class label $c$ and noise $z$ to each generated sample. The generator $G$ generates $X_{fake} = G(c,z)$ images, and the discriminator $D$ outputs a distribution of probability over class labels and sources, which can be represented as $P(S|X)$, $P(C|X) = D(X)$. The objective function of the ACGAN has two parts: the log-likelihood for the correct source, $L_{s}$, and the log-likelihood for the correct class, $L_{c}$, both given by the following equations:

2 $$Ls = E\left[ {\log P(S = real|X_{real} } \right] + E[\log P(S = fake|X_{fake} )],$$

3 $$L_{c} = E\left[ {\log P(C = c|X_{real} } \right] + E[\log P(C = c|X_{fake} )]$$

The discriminator is trained to maximize $L_{s}$ + $L_{c}$, and the generator is trained to maximize $L_{c}$—$L_{s}$^21^.

The generator network takes as input a latent vector of 100, drawn from a random normal distribution (standard deviation of 0.02) and the class label. It then outputs a maxillary sinus image of size 56 × 56 × 3. The class embedding of 50 dimensions is applied for categorical input. The network consists of three transposed convolutional layers [kernel of size (5, 5) and stride of (2, 2)], three ReLU activation layers, two batch normalization layers, and hyperbolic tangent (tanh) layers at the end of the model.

**Figure 5:**
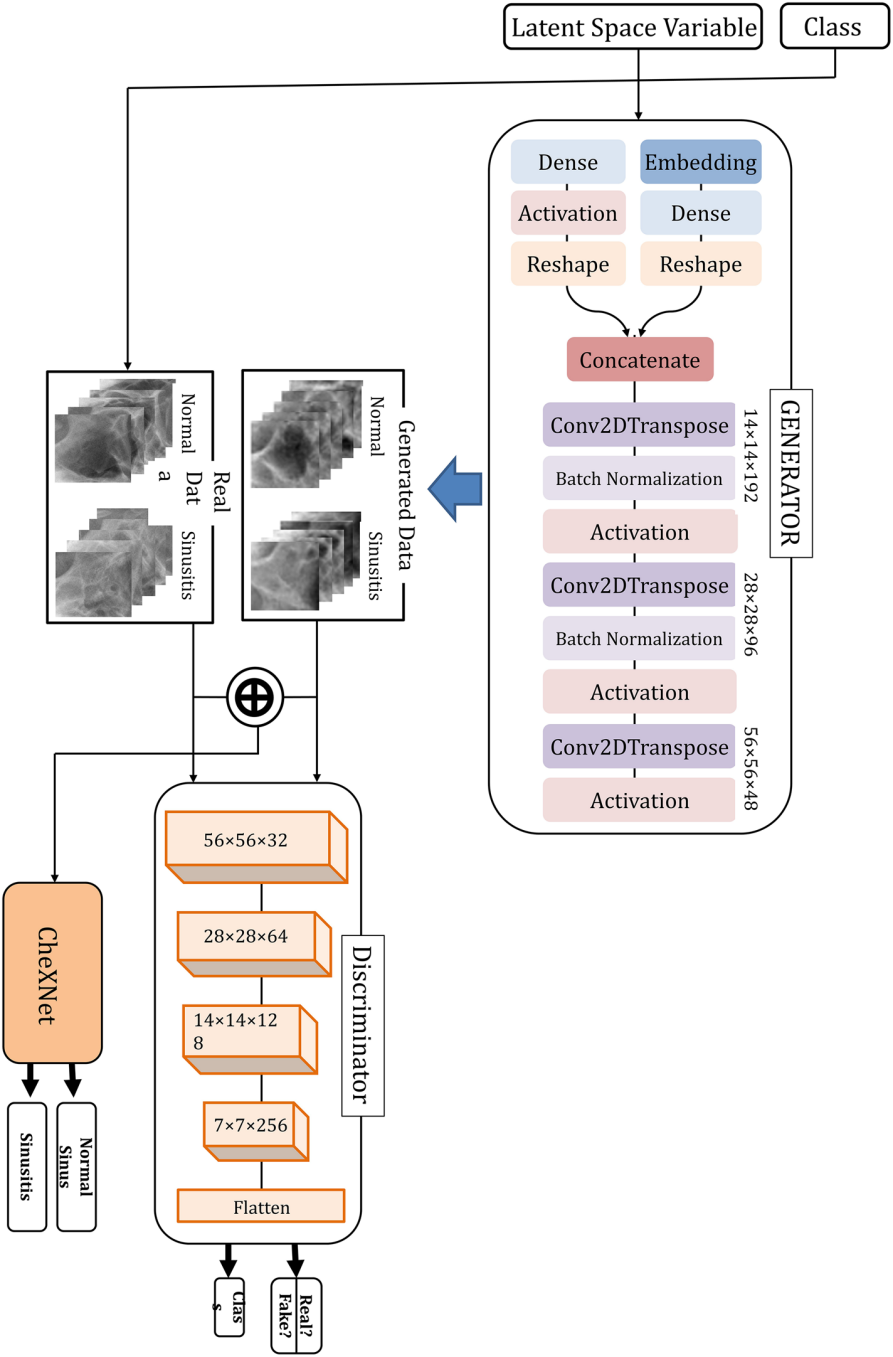
Auxiliary Classifier GAN (ACGAN) architecture and paranasal radiograph classification method.

The discriminator network takes an image of size 56 × 56 × 3 as the input. It then outputs a prediction if the image is real or fake, along with the class label corresponding to the prediction. The network consists of four convolutional layers [kernel of size (3, 3) and stride of (2, 2)], four LeakyReLU activation layers with a slope of 0.2, and four batch normalization layers. Every layer except the last one incorporates a dropout of 0.5. The final output is flattened, and the second layer uses the softmax function to predict the class label. The first output layer uses the flattened final output in the sigmoid function to predict the realness of the generated images. A detailed architecture of the ACGAN model is shown in Fig. 5.

**Figure 6:**
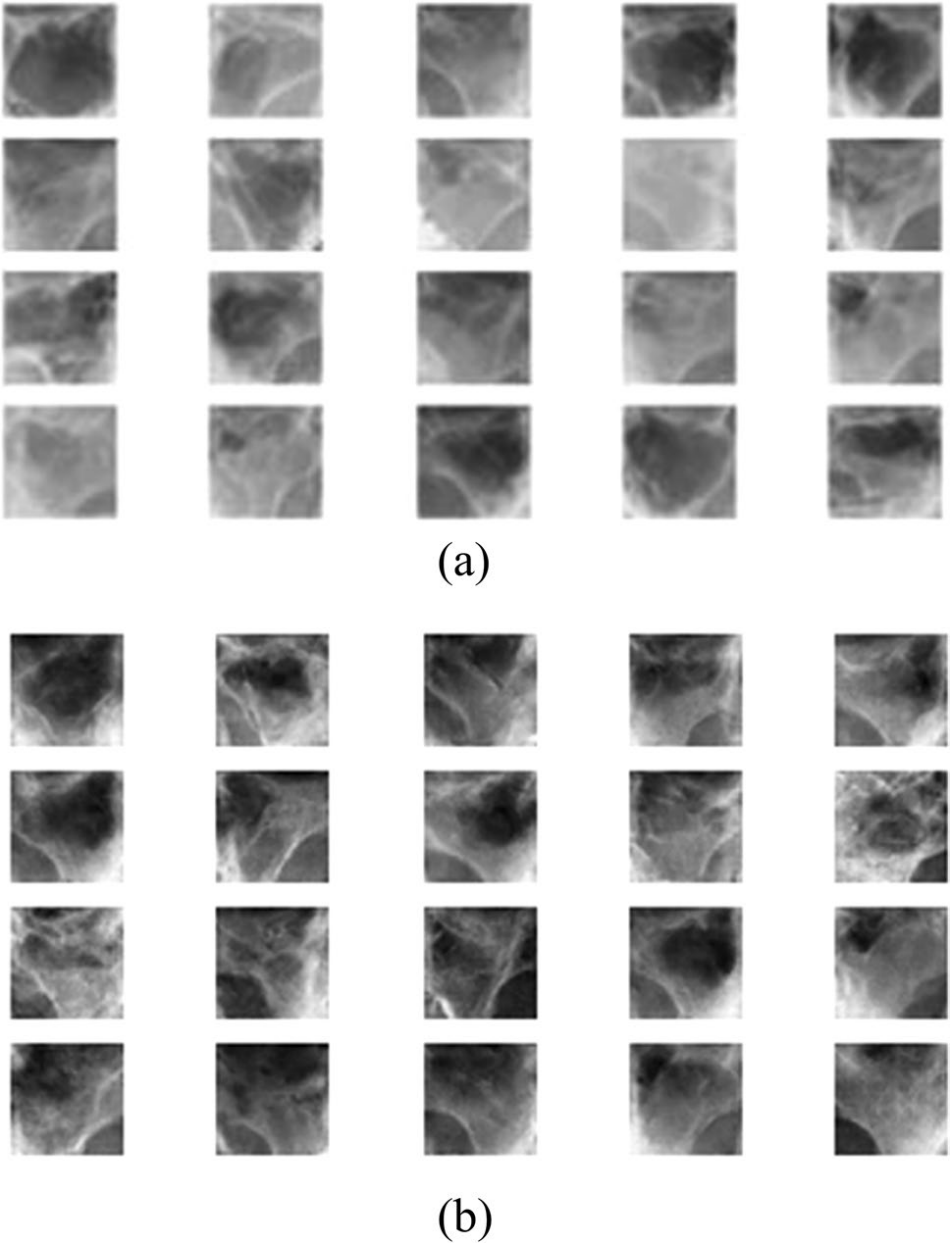
Clear sinus and sinusitis of maxillary sinuses. (**a**) Original images. (**b**) Synthetic images.

The following hyper-parameters are used for training ACGAN: Adam optimizer function with learning rate = 0.0002 and beta = 0.5, batch size = 64, epochs = 30,000. The training process of ACGAN is performed using an NVIDIA GeForce GTX 1050 Ti GPU. It takes approximately 5 h to train the model. Figure 6 shows the synthetic maxillary sinus images generated from ACGAN.

### Data augmentation methods

We used data augmentation to enlarge the training samples and improve the classification results for the maxillary sinusitis classification task. We augmented the data in two ways: (1) conventional data augmentation on pre-existing data samples; (2) synthesis of new samples learned from the original data samples using generative models. Class balancing using generated images by ACGAN was conducted beforehand to compare the effects of conventional data augmentation and GAN-based synthetic data augmentation.

#### Conventional data augmentation

**Figure 7:**
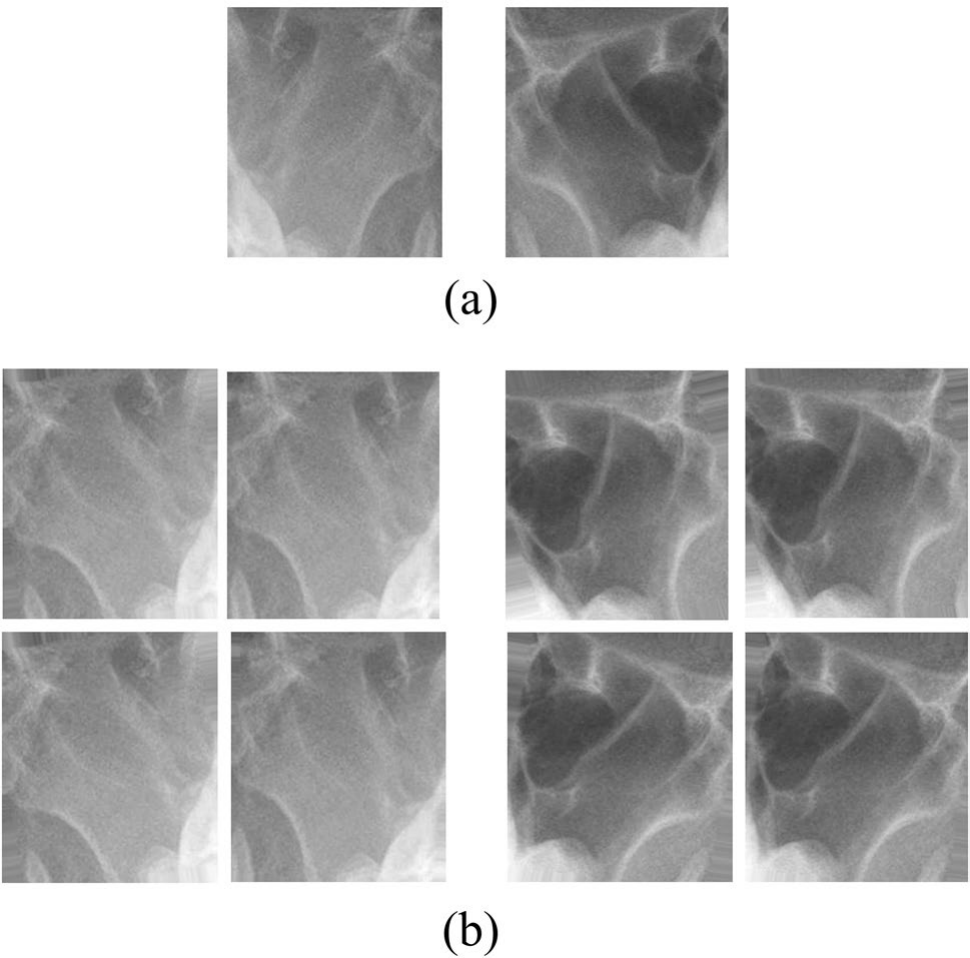
Samples of conventional data augmentation. (**a**) Horizontal flip. (**b**) Random rotation (− 10 to 10°).

As previously mentioned, the most common conventional data augmentation used for medical image analysis is geometric transformation (e.g., rotation, translation, shearing, etc.)^25^ and intensity transformation (e.g., blurring, adding noise, contrast, etc.). We applied geometric transformation for conventional data augmentation, which includes horizontal flip and rotation, as shown in Fig. 7. The rotation was conducted randomly within the range of − 10 to 10°. Both methods were applied to just the original images. We avoided transformations that cause shape deformation (e.g., shearing) to preserve the characteristics of the maxillary sinus.

#### Synthetic data augmentation

We loaded the generator weight of ACGAN to produce synthetic data and subsequently combined the generated synthetic maxillary sinus images with the pre-existing training set, which consisted of original data. Because we did not know the ideal amount of synthetic data that was needed, we investigated the effect of increasing the amount of synthetic data on the performance of the classification model.

The sliding window method was applied to find the optimal point of synthetic data augmentation at the highest diagnostic performance of the model, which we refer to as the optimal multiple number (K_optimal_). The sliding window analyzes the given array using a window that is in the form of a subarray of size *w*. The maximum value within the window size is printed each time the window shifts from left (old data) to the right (new data)^26^.

In the present study, the sliding window size, *w,* was heuristically selected as 12. The subarray values are the AUC scores (user’s selection). The synthetic data augmentation starts with the numbers of multiple 1 (K = 1) for the training set and increases by 1 as the automation process iterates. The sliding window starts when the model training iterates more than the number of the window size, *w*. The index of the *AUC_array* is shifted by 1 at each iteration during the sliding window process, and the whole process stops when the optimal multiple number (K_optimal_) is found. The accumulated trained model of each multiple number (K) is then selected corresponding to the final optimal multiple number (K_optimal_).

**Figure 8:**
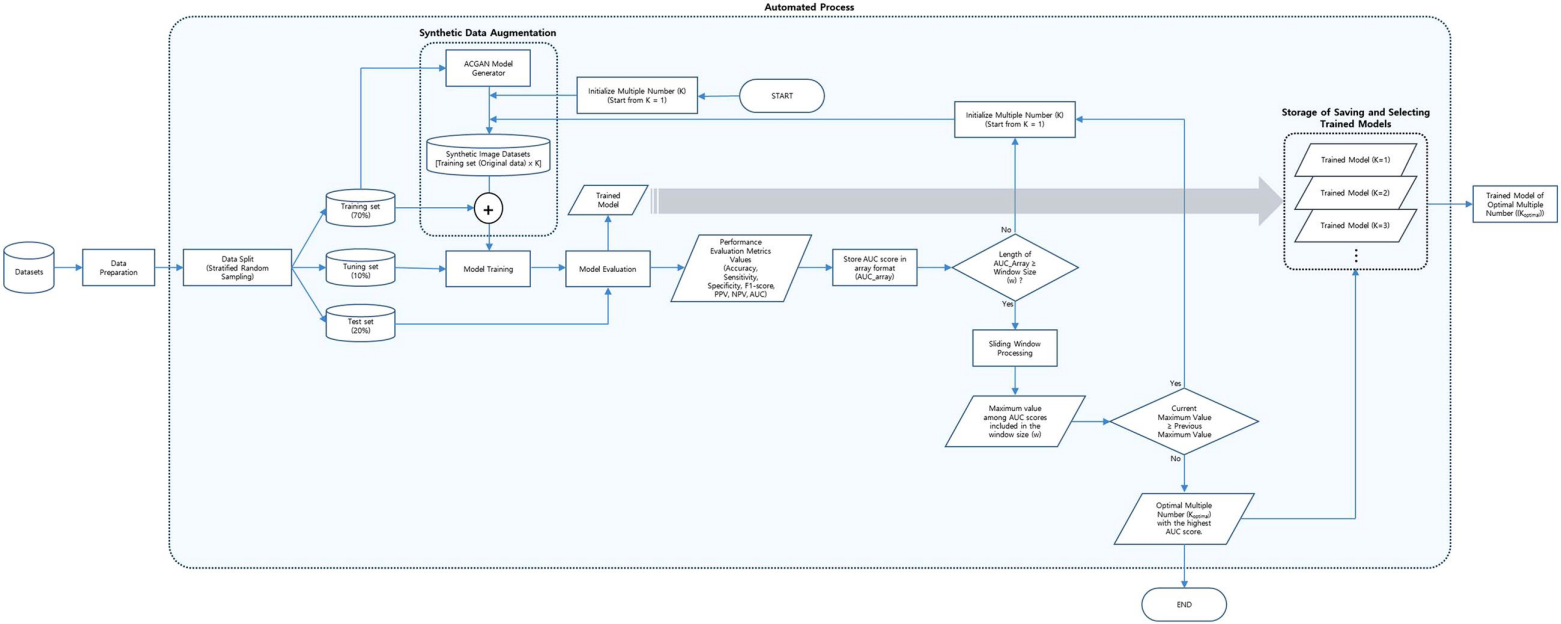
Flowchart describing the automation pipeline of GAN-based synthetic data augmentation process to find the optimal multiple number at the highest model performance.

The classification model was trained using all possible numbers of the multiples, and its diagnostic performance was checked each time. By doing so, we looked for K_optimal_ to be used for selecting the best-trained model with the highest diagnostic performance. The overall automation pipeline is represented in Fig. 8, and the details of the sliding window phase can be seen in Supplementary Fig. S3.

### Classification of PNS X-ray

The transfer learning method was used with the CheXNet^20^ model to diagnose maxillary sinusitis. Transfer learning is a technique that enables learning new problems by using a model that has already been trained using large datasets. The CheXNet model was chosen because radiographs of the chest and PNS have similar imaging characteristics. The pre-trained model weight of CheXNet was loaded and transferred to train on our PNS radiographs. The patch images were resized to 56 × 56 pixels using bilinear interpolation in the training procedure. The pre-trained CheXNet model weights were fixed for the feature extractor. The last layer of the fully connected layers was modified to output two categories: normal sinus and sinusitis. The model was tuned with the following hyperparameters: learning rate = 0.0001, batch size = 8, epochs = 100, binary cross entropy as loss function, and Adam optimizer with an initial learning rate of 0.0001. The decays by the given initial learning rate were divided using the number of epochs. The training process of CheXNet was performed on a deep learning server with a Tesla K80 GPU (NVIDIA, Santa Clara, California).

### Statistical analysis

The accuracy, sensitivity, specificity, F1-score, positive predictive value (PPV), and negative predictive value (NPV) were calculated^27^ to evaluate the classification performance of PNS radiographs. The AUC and receiver operating characteristic (ROC) curves were also calculated. The evaluation was done with the fixed internal and external test set. All performances were calculated using the default cutoff value of 0.5.

A DeLong Test was used to compare the diagnostic performances between the models, and a *P* value less than 0.05 was considered statistically significant. Statistical analyses were performed using R for Windows ver. 4.0.3 (R Foundation for Statistical Computing, Vienna, Austria) and IBM SPSS Statistics software ver. 26.0 (IBM Corp., Armonk, NY, USA).

## Supplementary Information

Supplementary Information.

## Data Availability

The original datasets of this study are available on reasonable request due to patient privacy issues. The synthetic data generated using ACGAN in this study are available on a public repository: https://github.com/hmoonGAN/GAN_proj.
