# Supplementary material for: Automation of generative adversarial network-based synthetic data-augmentation for maximizing the diagnostic performance with paranasal imaging
Source: Sci Rep. 2022 Oct 27;12:18118. doi: 10.1038/s41598-022-22222-z (PMC9613909; doi:10.1038/s41598-022-22222-z)
Supplement: Supplementary file 1 — Supplementary Information. [file 41598_2022_22222_MOESM1_ESM.docx]

**SUPPLEMENTAL MATERIALS**

Automation of Generative Adversarial Network-based Synthetic Data-Augmentation for Maximizing the Diagnostic Performance with Paranasal Imaging

Hyoun-Joong Kong^+^, Jin Youp Kim^+^, Hye-Min Moon^+^, Hae Chan Park, Jeong-Whun Kim, Ruth Lim, Jonghye Woo, Georges El Fakhri, Dae Woo Kim*, and Sungwan Kim*

**SUPPORTING INFORMATION**

**Clinical Explanation on Datasets based on Paranasal Sinus X-Ray**

In the present study, deep learning methods were applied for the diagnosis of maxillary sinusitis. Sinusitis is a relatively common condition characterized by inflammation of the mucosal lining of paranasal sinuses [PNSs] [1]. Sinusitis can involve all sinuses. However, the maxillary sinus is the most commonly affected sinus. Maxillary sinusitis is usually diagnosed by the opacification and air/fluid level of the sinuses on conventional radiographs or computed tomography (CT). X-ray images are still used for the initial diagnosis of sinusitis instead of CT scans owing to their affordability and lower radiation exposure [2,3].

Supplementary Table S1. Performance evaluation of internal test set for the CheXNet [4] model with the increase in training set size.

| Number of Training Images (+synthetic images) | Accuracy | Sensitivity | Specificity | F1-score | PPV^a^ | NPV^b^ | AUC^c^ |
| --- | --- | --- | --- | --- | --- | --- | --- |
| ×2 (n=608) | 0.81 | 0.767 | 0.861 | 0.815 | 0.868 | 0.756 | 0.871 |
| ×3 (n=912) | 0.823 | 0.837 | 0.806 | 0.837 | 0.837 | 0.806 | 0.901 |
| ×4 (n=1,216) | 0.81 | 0.744 | 0.889 | 0.81 | 0.889 | 0.744 | 0.915 |
| ×5 (n=1,520) | 0.86 | 0.884 | 0.833 | 0.874 | 0.864 | 0.857 | 0.911 |
| ×6 (n=1,824) | 0.848 | 0.847 | 0.861 | 0.857 | 0.878 | 0.816 | 0.896 |
| ×7 (n=2,128) | 0.861 | 0.884 | 0.833 | 0.874 | 0.864 | 0.857 | 0.92 |
| ×8 (n=2,432) | 0.873 | 0.884 | 0.861 | 0.884 | 0.884 | 0.861 | 0.944 |
| ×9 (n=2,736) | 0.861 | 0.861 | 0.861 | 0.871 | 0.881 | 0.838 | 0.941 |
| ×10 (n=3,040) | 0.861 | 0.884 | 0.833 | 0.874 | 0.864 | 0.857 | 0.912 |
| ×11 (n=3,344) | 0.848 | 0.814 | 0.889 | 0.854 | 0.897 | 0.8 | 0.937 |
| ×12 (n=3,648) | 0.835 | 0.837 | 0.833 | 0.847 | 0.857 | 0.811 | 0.914 |
| ×13 (n=3,952) | 0.886 | 0.93 | 0.833 | 0.899 | 0.87 | 0.909 | 0.939 |
| ×14 (n=4,256) | 0.899 | 0.907 | 0.889 | 0.907 | 0.907 | 0.889 | 0.963 |
| ×15 (n=4,560) | 0.861 | 0.861 | 0.861 | 0.871 | 0.881 | 0.838 | 0.922 |
| ×16 (n=4,864) | 0.911 | 0.907 | 0.917 | 0.918 | 0.929 | 0.892 | 0.959 |
| ×17 (n=5,168) | 0.861 | 0.814 | 0.917 | 0.864 | 0.921 | 0.805 | 0.94 |
| ×18 (n=5,472) | 0.874 | 0.861 | 0.889 | 0.881 | 0.902 | 0.842 | 0.928 |
| ×19 (n=5,776) | 0.886 | 0.884 | 0.889 | 0.864 | 0.905 | 0.865 | 0.957 |
| ×20 (n=6,080) | 0.899 | 0.907 | 0.889 | 0.907 | 0.907 | 0.889 | 0.946 |
| ×21 (n=6,384) | 0.84 | 0.837 | 0.833 | 0.847 | 0.857 | 0.811 | 0.939 |
| ×22 (n=6,688) | 0.871 | 0.855 | 0.889 | 0.874 | 0.894 | 0.849 | 0.926 |
| ×23 (n=6,992) | 0.823 | 0.814 | 0.833 | 0.833 | 0.854 | 0.799 | 0.93 |
| ×24 (n=7,296) | 0.75 | 0.646 | 0.889 | 0.747 | 0.886 | 0.653 | 0.92 |
| ×25 (n=7,600) | 0.861 | 0.884 | 0.833 | 0.874 | 0.864 | 0.857 | 0.928 |
| ×26 (n=7,904) | 0.861 | 0.861 | 0.861 | 0.871 | 0.881 | 0.838 | 0.933 |

^a^PPV = Positive predictive value

^b^NPV = Negative predictive value

^c^AUC = Area under the curve


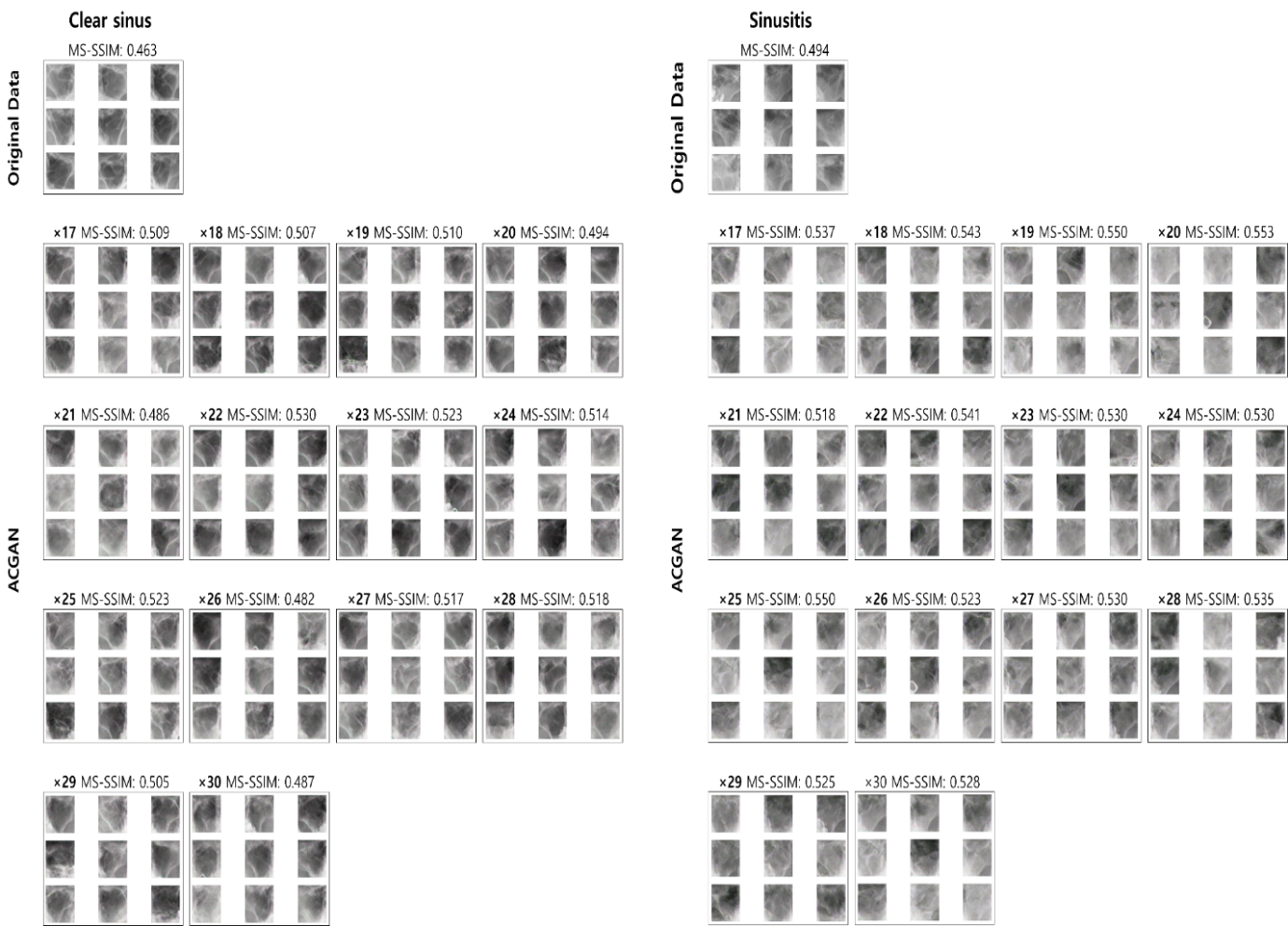


Supplementary Figure S2. MS-SSIM [6] scores of randomly chosen 100 image patches of the clear sinus (left) and sinusitis (right) image pairs for original data and data generated from the Auxiliary Classifier Generative Adversarial Network (ACGAN) [7].


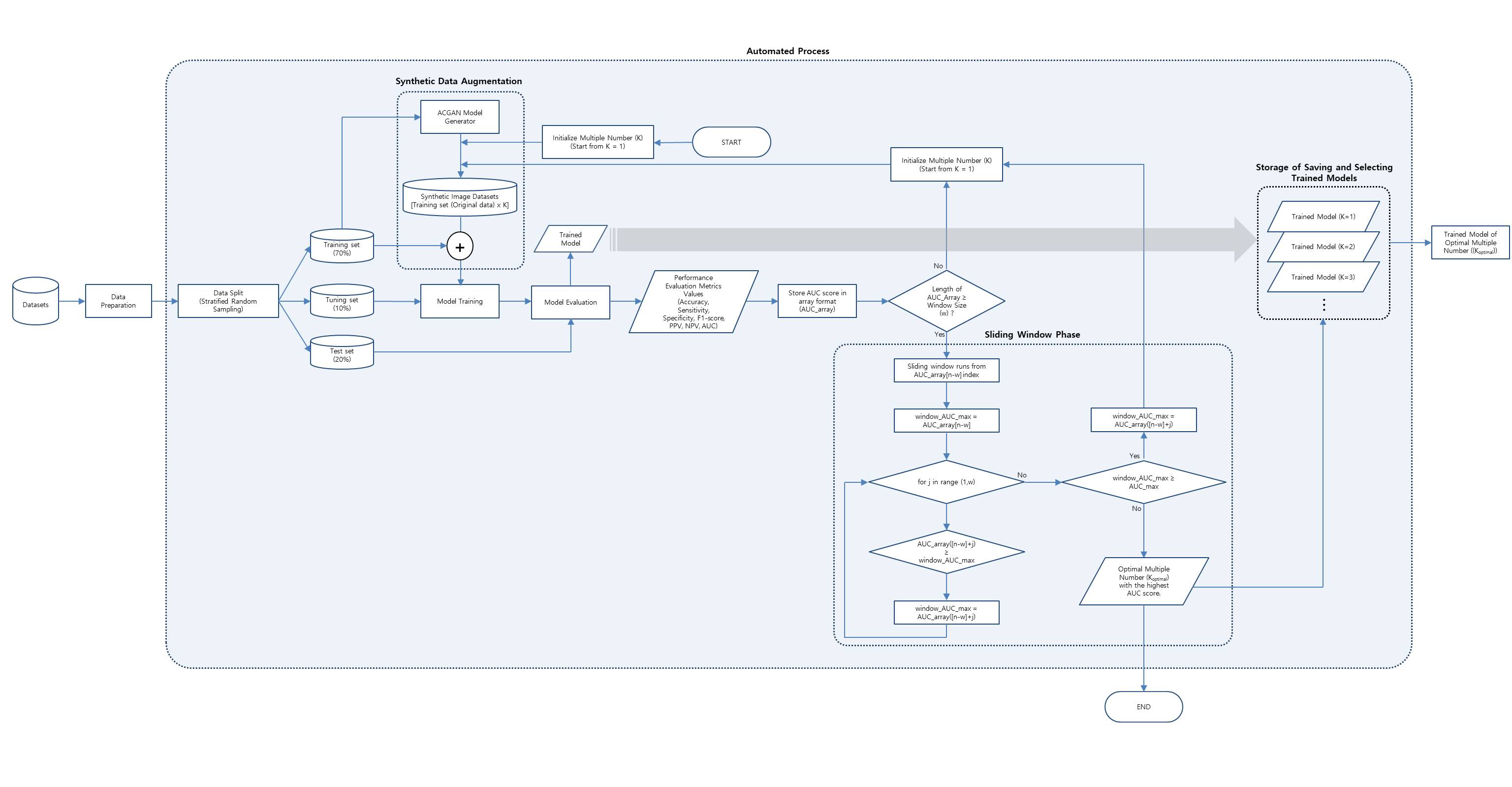


**Supplementary Figure S3**. The sliding window starts from the index ([n-w]) of AUC_array, finds the maximum values in w, and updates it into a local variable, window_AUC_max. The old window_AUC_max value and the new window_AUC_max values are compared during each iteration. If the new window_AUC_max is the same or bigger than the previous value, it is updated to AUC_max and runs the next iteration for synthetic data augmentation by increasing the number of multiples by 1 (i.e., K = K+1). Whenever the sequence of maximum values (AUC_max) of the single w shows a decrease, the sliding window stops and finally outputs the optimal multiple number (K_optimal_). The trained model of K_optimal_ (M_optimal_) is then selected according to the value of (K_optimal_) (n: the length of AUC_array; w: sliding window size).

**References**

1. Helliwell, T. Inflammatory diseases of the nasal cavities and paranasal sinuses. *Diagn Histopathol (Oxf)*. **16,** 255-264 (2010).
2. Okuyemi, K. S. & Tsue, T. T.  Radiologic imaging in the management of sinusitis. *Am. Fam. Physician*. **66,** 1882-1886 (2002).
3. Burke, T. F., Guertler, A. T. & Timmons, J. H. Comparison of sinus x-rays with computed tomography scans in acute sinusitis. *Acad. Emerg. Med*. **1,** 235-239 (1994).
4. Rajpurkar, P. et al. CheXNet: Radiologist-level pneumonia detection on chest X-rays with deep learning. Available: arxiv.org/abs/1711.05225 (2017).
5. Wang, Z., Simoncelli, E. P. & Bovik, A. C. Multiscale structural similarity for image quality assessment. *IEEE Asilomar Conference on Signals, Systems, and Computers.* **2,** 1398-1402 (2003).
6. Odena, A., Olah, C., & Shlens, J. Conditional image synthesis with auxiliary classifier GANs. *Proceedings of the 34th International Conference on Machine Learning*. **70,** 2642-2651 (2017).
